# Supplementary material for: WT1-AS/IGF2BP2 Axis Is a Potential Diagnostic and Prognostic Biomarker for Lung Adenocarcinoma According to ceRNA Network Comprehensive Analysis Combined with Experiments
Source: Cells. 2021 Dec 23;11(1):25. doi: 10.3390/cells11010025 (PMC8750352; doi:10.3390/cells11010025)
Supplement: Supplementary file 1 [file cells-11-00025-s001.zip › cells-1454751-supplementary.pdf]

Supplementary Material

Table S1. IHC of c-Myc in Human Protein Atlas database.

No.	Gene	Tissue type	ID	Age	Gender	staining
1	c-Myc	Normal	705	61	Female	Low
2	c-Myc	Normal	218	73	Male	Low
3	c-Myc	Adenocarcinoma	1327	64	Male	Medium
4	c-Myc	Adenocarcinoma	1249	44	Female	High

Table S2. Univariate analysis of overall survival in LUAD patients stratified based on clinical characteristics.

Factor	Variable	N	WT1-AS Expression (Median)	P -value	miR-200a-3p Expression (Median)	P -value	IGF2BP2 Expression (Median)	P -value	Overall survival		
									Months (mean)	95% CI (mean)	P-value (log-rank test)
Age	>60	333	4	0.5059	3021	0.6062	830	0.5306	32.127	25.26–30.53	0.3639
	≤60	154	3		3971		895		27.893	26.09–38.17	
Gender	Male	232	5	0.4954	3309.5	0.4809	985.5	0.1820	30.523	26.19–34.87	0.9138
	Female	265	4		3498		801		29.86	26.63–33.09	
Tumor size (T)	>3	328	5	0.0449	3462.5	0.8975	975.5	0.0434	30.907	26.66–33.14	0.0048
	≤3	166	3		3317		735		29.9	25.6–35.6	
Lymph-node metastasis (N)	Negative	320	4	0.3920	3567	0.0474	869	0.8683	32.543	28.98–36.1	<0.0001
	Positive	165	4		2991		890		25.97	22.08–29.86	
Distant metastasis (M)	Negative	330	4.5	0.1483	3259	0.7043	796	0.8538	31.813	28.49–35.13	0.0023
	Positive	23	2		2991		978		24.207	16.01–32.4	
TNM stage	I–II	386	5	0.6111	3700.5	0.0026	796	0.0464	31.85	28.69–35	<0.0001
	III–IV	104	2.5		2493		1067.5		23.083	18.94–27.23	

Table S3. Univariate and multivariate analysis (Cox regression model) of WT1-AS and IGF2BP2 in LUAD patients.

**Factor**	**Univariate Cox**			**Munivariate Cox**		
	**HR**	**95% CI**	**P value (Log-rank test)**	**HR**	**95% CI**	**P value (Log-rank test)**
Age	0.952	0.729–1.245	0.722			
Gender	0.972	0.726–1.303	0.851			
TNM stage	1.758	1.364–2.266	<0.0001	1.341	1.009–1.784	0.043
Tumor size (T)	1.659	1.178–2.336	0.004	1.456	1.029–2.059	0.034
Lymph node metastasis (N)	2.035	1.598–2.592	<0.0001	1.818	1.393–2.374	<0.0001
Distant metastasis (M)	0.951	0.801–1.129	0.564			
WT1-AS expression (high/low)	1.229	0.917–1.647	0.168			
IGF2BP2 expression (high/low)	1.262	0.942–1.692	0.119			

Table S4. Correlation analysis between IGF2BP2 and biomarkers of immune cells using TIMER.

Description	Gene markers	LUAD	
		Cor	p-value
CD8+ T cell	CD8A	0.172	8.56×10^{-5}
	CD8B	0.156	3.77×10^{-4}
T cell (general)	CD3D	0.114	9.66×10^{-3}
	CD3E	0.12	6.38×10^{-3}
	CD2	0.1	2.35×10^{-5}
B cell	CD19	0.021	6.36×10^{-1}
	CD79A	0.02	6.54×10^{-1}
Monocyte	CD86	0.161	2.45×10^{-4}
	CD115 (CSF1R)	0.17	1.04×10^{-4}
TAM	CCL2	0.163	2.01×10^{-4}
	CD68	0.177	5.49×10^{-5}
	IL10	0.123	5.13×10^{-3}
M1 Macrophage	INOS (NOS2)	0.153	5.12×10^{-4}
	IRF5	0.212	1.18×10^{-6}
	COX2 (PTGS2)	0.099	2.49×10^{-5}
M2 Macrophage	CD163	0.197	6.81×10^{-6}
	VSIG4	0.106	1.63×10^{-2}
	MS4A4A	0.093	3.39×10^{-2}
Neutrophils	CD66b (CEACAM8)	−0.122	5.57×10^{-03}
	CD11b (ITGAM)	0.175	6.30×10^{-05}
	CCR7	0.071	1.08×10^{-01}
Natural killer cell	KIR2DL1	0.051	2.46×10^{-01}
	KIR2DL3	0.147	8.21×10^{-04}
	KIR2DL4	0.239	4.09×10^{-08}
	KIR3DL1	0.082	6.24×10^{-02}
	KIR3DL2	0.104	1.78×10^{-02}
	KIR3DL3	0.108	1.44×10^{-02}
	KIR2DS4	0.132	2.59×10^{-03}
Dendritic cell	HLA-DPB1	−0.101	2.25×10^{-02}

	HLA-DQB1	−0.075	9.09×10^{-02}
	HLA-DRA	−0.079	7.46×10^{-02}
	HLA-DPA1	−0.056	2.09×10^{-01}
	BDCA-1 (CD1C)	−0.151	5.82×10^{-04}
	BDCA-4 (NRP1)	0.161	2.42×10^{-04}
	CD11c (ITGAX)	0.158	3.09×10^{-04}
Th1	T-bet (TBX21)	0.205	2.84×10^{-06}
	STAT4	0.044	3.23×10^{-01}
	STAT1	0.373	2.95×10^{-18}
	TNF-γ (IFNG)	0.183	2.89×10^{-05}
	TNF-α (TNF)	0.114	9.62×10^{-03}
Th2	GATA3	0.23	1.25×10^{-07}
	STAT6	0.012	7.88×10^{-01}
	STAT5A	0.245	1.86×10^{-08}
	IL13	0.04	3.71×10^{-01}
Tfh	BCL6	0.079	7.28×10^{-02}
	IL21	0.143	1.10×10^{-03}
Th17	STAT3	0.065	1.39×10^{-01}
	IL17A	0.045	3.09×10^{-01}
Treg	FOXP3	0.196	7.73×10^{-06}
	CCR8	0.186	2.12×10^{-05}
	STAT5B	0.22	4.34×10^{-07}
	TGFβ (TGFB1)	0.186	2.07×10^{-05}
T cell exhaustion	PD-1 (PDCD1)	0.259	1.50×10^{-07}
	CTLA4	0.184	2.73×10^{-05}
	LAG3	0.217	4.94×10^{-08}
	TIM-3 (HAVCR2)	0.159	2.86×10^{-04}
	GZMB	0.278	1.43×10^{-10}

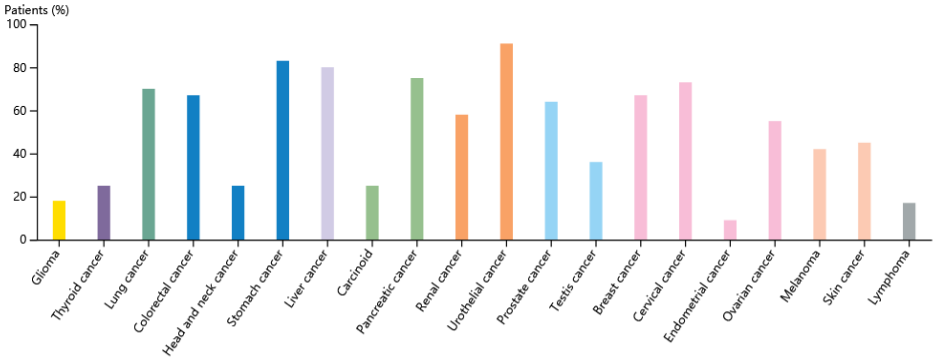

Figure S1. Expression distribution expression of c-Myc in various cancer tissues.

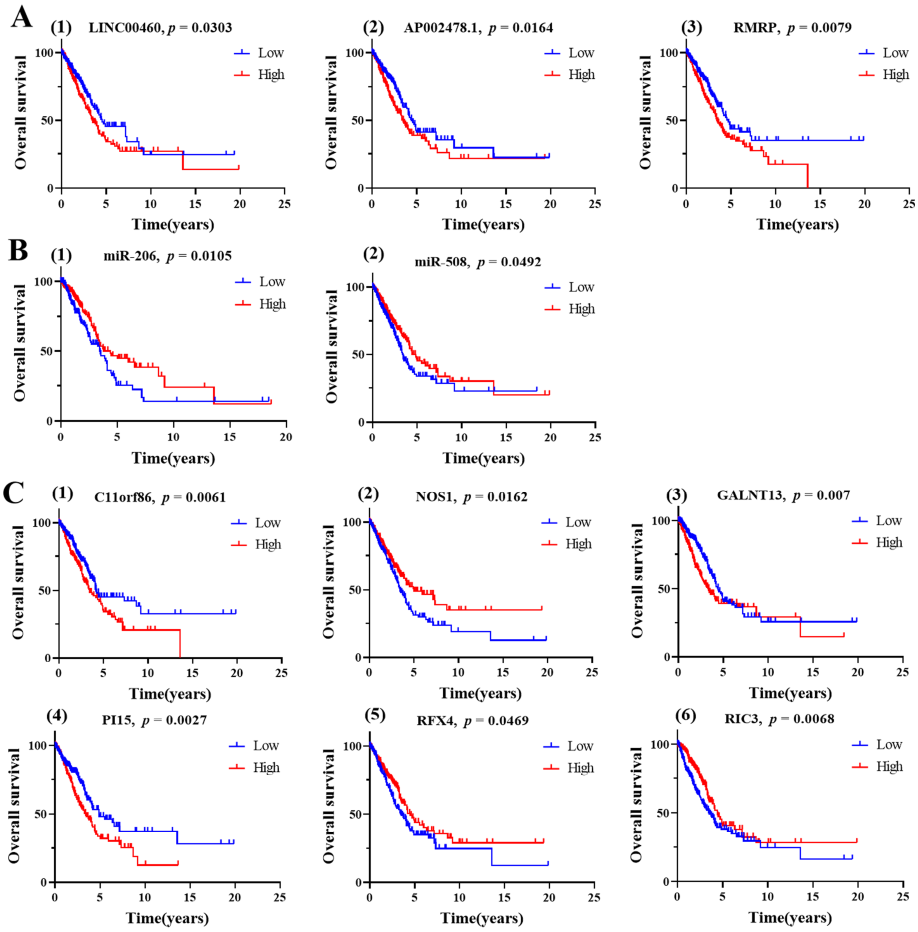

Figure S2. Correlation analysis of differentially expressed RNAs in ceRNA network with overall survival of LUAD patients. The high-expression and low-expression value of (A) 4 lncRNAs, (B) 3 miRNAs and (C) 8 mRNAs were compared by Kaplan–Meier survival curve for LUAD patient cohort. P<0.05 was deemed as statistically significant.

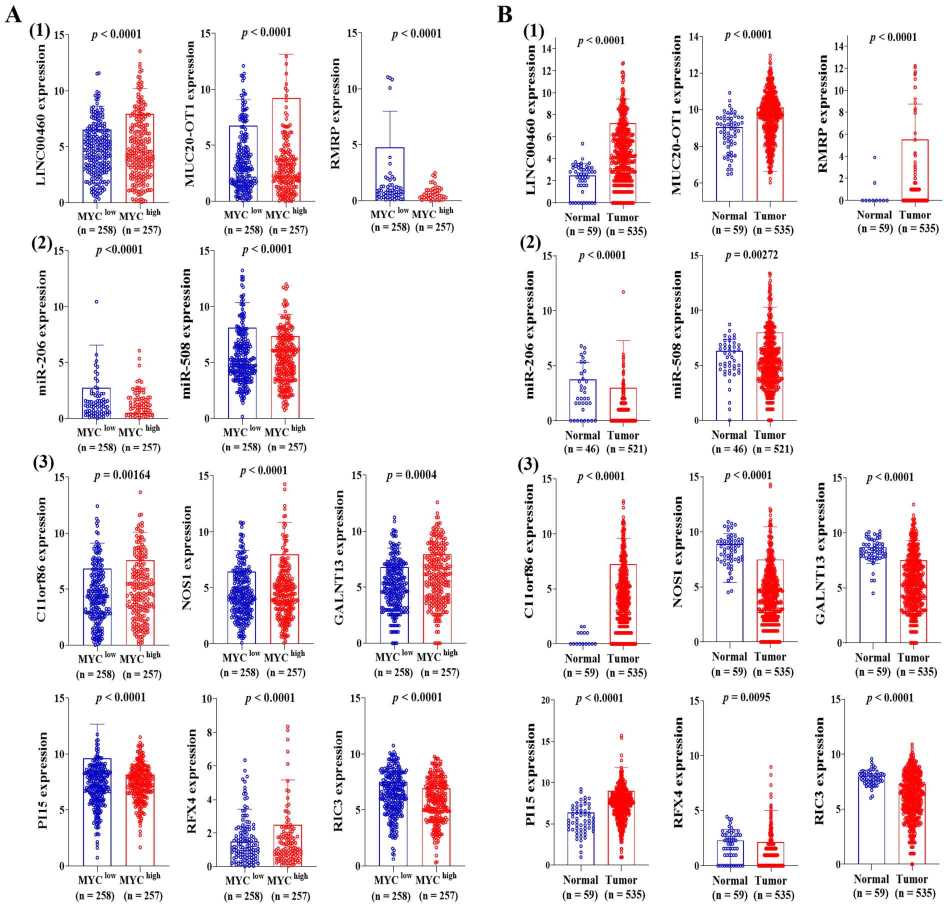

Figure S3. The distribution of 15 RNAs expression value from the ceRNA network in the TCGA LUAD dataset. The expression pattern of 4 lncRNAs, 3 miRNAs and 8 mRNAs in LUAD samples with c-Myc^{high} and c-Myc^{low} groups (**A**) as well as in LUAD and adjacent-normal lung tissues (**B**). The RNAs expression value was logarithmized with log2. P<0.05 was deemed as statistically significant.

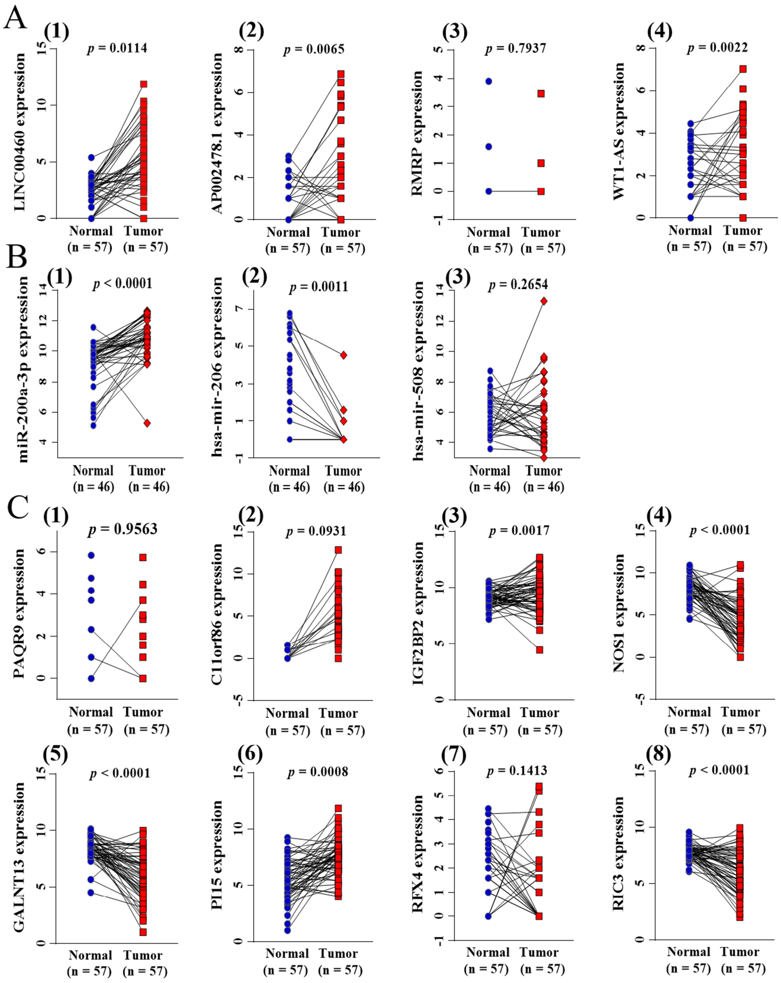

Figure S4. The distribution of 15 RNAs expression value in paired LUAD tissues.

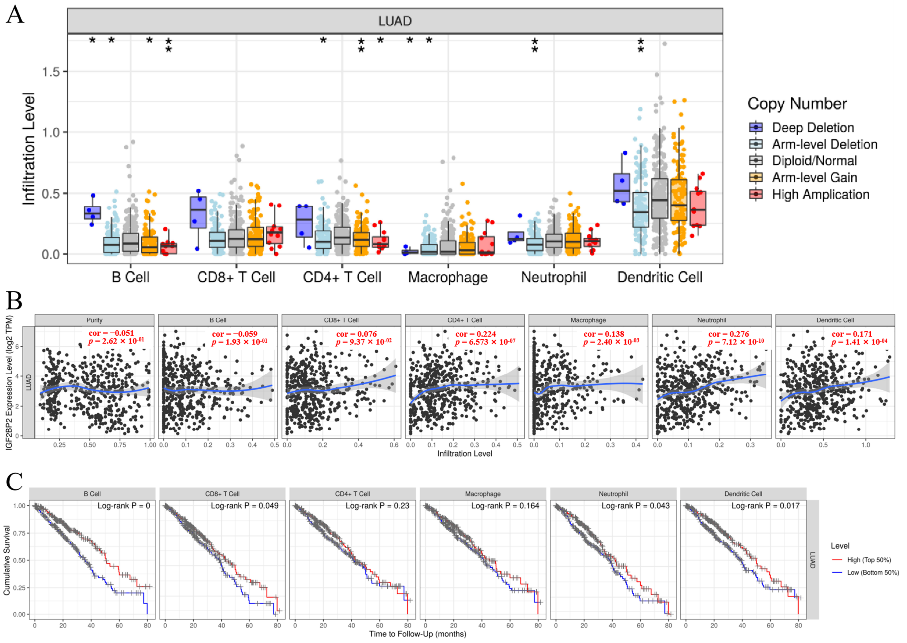

Figure S5. Correlation analysis of IGF2BP2 expression levels with immune infiltration in lung adenocarcinoma. (A) Correlation between IGF2BP2 gene copy numbers and the level of infiltration of six immune cells in LUAD. (B) Correlation between IGF2BP2 expression and the level of immune infiltration in LUAD. (C) Correlation analysis of immune infiltration and OS of LUAD. * P < 0.05; ** P < 0.01. P<0.05 was deemed as statistically significant.
